# An instrument for measuring the influence of nursing care on the length of stay in heart failure hospitalizations of African Americans

**DOI:** 10.1017/cts.2026.10700

**Published:** 2026-02-04

**Authors:** Tremaine Brueon Williams, Milan Bimali, Pearman Parker, Alisha Crump, Emel Seker, Maryam Y. Garza, Melody Greer, Taren Massey-Swindle, Kevin Wayne Sexton

**Affiliations:** 1 Biomedical Informatics, University of Arkansas for Medical Sciences, USA; 2 Biostatistics, University of Arkansas for Medical Sciences, USA; 3 College of Nursing, University of Arkansas for Medical Sciences, USA; 4 Department of Population Health Sciences, The University of Texas Health Science Center at San Antonio, USA; 5 Department of Pediatrics, University of Arkansas for Medical Sciences, USA; 6 Arkansas Children’s Research Institute, Arkansas Children’s Nutrition Center, USA; 7 Department of Surgery, Vanderbilt University Medical Center, USA; 8 Department of Biomedical Informatics, Vanderbilt University Medical Center, USA

**Keywords:** Sociotechnical, human factors, electronic health records, registered nurses, congestive heart failure

## Abstract

**Introduction:**

The impact of guideline-directed medical therapy (GDMT) has not fully translated to decreases in the disproportionate rates of hospitalization and lengths of stay in African Americans with congestive heart failure (CHF). GDMT is optimized by registered nurses (RNs) and their use of clinical information. Yet, there are no instruments for measuring the influence of clinical information use and nursing care. The study assessed an instrument’s ability to measure the influence of RN performance of social, technical, and socio-technical care tasks on length of stay in the CHF hospitalizations of African Americans.

**Methods:**

A sample of 200 RNs, who cared for 5060 African Americans with 14,123 heart failure hospitalizations, were surveyed. Descriptive statistics, Cronbach’s alpha, and a generalized linear regression assessed the instrument’s reliability and predictive validity.

**Results:**

The Cronbach’s alpha was 0.95 (95% CI: 0.94–0.96). The corrected item-total correlations for the 22 items ranged from 0.44 to 0.80. For an increase of one to four points per item in a RN’s performance, the estimated reductions in the patient’s length of stay were 3.34% (6.11,0.5), 6.58% (11.84,1), 9.70% (17.22,1.49), and 12.72% (22.28,1.99), respectively (*P* = 0.004).

**Conclusions:**

Increases in a RN’s performance of social, technical, and socio-technical care tasks were significantly associated with clinically meaningful decreases in their patients’ length of stay. The instrument has strong potential for addressing the disproportionate impact of CHF by measuring and tailoring interventions to optimize nursing care and the use of clinical information in the provision and receipt of GDMT.

## Introduction

Approximately 6.7 million adults in the United States have congestive heart failure (CHF), and there is a projected increase in prevalence to 8 million by 2030 [1]. National trends on CHF hospitalizations have revealed the burdensome impact of CHF on African Americans. Compared to Caucasians, African Americans have the highest age-adjusted death rate, a 2.5 times greater hospitalization rate, and a fourth of a day longer length of hospital stay [2–5]. These outcomes have been exacerbated by the complex influences of systems of care (e.g., providers, patients, technologies) in the provision and receipt of guideline-directed medical therapy (GDMT) [5–7].

GDMT is a set of structured clinical practice guidelines for clinical evaluation, diagnostic testing, and targeted procedural and pharmacological treatments for CHF [6–10]. National quality improvement initiatives such as the American Heart Association’s Get with the Guidelines for Heart Failure (GWTG-HF) facilitate the implementation and reporting of performance metrics related to GDMT at hospitals in the United States [8,9,11]. GWTG-HF provides toolkits to mitigate the negative influences of systems of care. This includes facilitating the evaluation of left ventricular function, the prescribing of evidence-based medications, providing pre-discharge CHF education for self-care, coordinating processes such as scheduling follow-up visits within 72 hours of discharge, and providing pre-discharge assessment of social determinants of health barriers [11]. GDMT has advanced the use of evidence-based clinical practice in African Americans through, for example, standardizing the use of beta blocker-hydralazine nitrate combinations at discharge for targeted therapy [6–9]. However, the subsequent translation of GDMT and its impact on rates of hospitalization and lengths of stay have been inhibited by additional system factors such as clinical information use, which can constrain the provision and receipt of GDMT and the quality of care in African Americans [9–12]. For example, the ability of a Registered Nurse (RN) to work with clinical information related to a patient’s CHF in the electronic health record (EHR) system could prompt or constrain changes in specific CHF orders from physicians [13]. Recently, these system factors have been embedded within national guidelines for the management of heart failure, specifically calling for research to identify the characteristics of EHR use that optimize the provision and receipt of GDMT [9]. These directives specifically noted a need to strengthen the evidence base of how clinicians perform EHR tasks and the related care processes for supporting real-time feedback on performance measures that impact patient outcomes, such as lengths of stay [9,12–16].

Among front-line clinicians, RNs are responsible for executing many of the routine, high-frequency EHR tasks that facilitate the delivery of effective GDMT [14]. RNs, and their performance of these social, technical, and socio-technical care delivery tasks, have been significantly associated with a 30% decrease in hospitalization rates, a 28%–31% decrease in 30-day readmission odds over 7 years, and a 9%–85% decrease in odds of long lengths of stay [13–17]. Care tasks performed by RNs include social tasks (e.g., giving patient education information to patients or their caregivers), technical tasks (e.g., accessing clinical resources to care for their CHF patients), and socio-technical tasks (e.g., risk-stratifying CHF patients based on risk scores in the EHR) [13,17]. These recent findings and research directives substantiate the need to develop an instrument for measuring the influence of clinical information use and nursing care on patients.

The current validated instruments and tools aim to optimize length of stay by measuring, risk-stratifying, predicting, and controlling for the influence of seven types of variables, ranging from demographic to clinical (Table 1) [9,13,18–31]. However, in excluding the influence of RNs and how they use clinical data to care for the patient, the current instruments and tools have had only moderate success in comprehensively approaching decreases in lengths of stay [16]. Fifteen validated instruments have been designed to specifically measure task performance of RNs, but lack a focus on decreasing rates of hospitalization and lengths of stay [30,31]. Of the fifteen instruments, eight were designed for students and three were designed for nurse leaders [30,31]. Three of the remaining four instruments focus on bedside nurses: the Nursing Informatics Competency Assessment Tool, Nursing Informatics Competency Assessment L3/L4, and the TIGER-Based Assessment of Nursing [30,31]. These three instruments were further limited by the exclusion of items that assessed tasks specific to basic chronic care delivery and the unique burden of social determinants of health on African Americans. However, the remaining instrument, the Hennessey-Hicks Training Need Analysis, is focused on bedside nursing, basic chronic care delivery, and can be adapted to measure elements of care that are unique to African Americans with CHF, such as the burden of social determinants of health [31]. The instrument is endorsed by the World Health Organization and globally applied in healthcare quality initiatives and clinical practice to assess the performance of RNs and other clinicians. Thirty-three prior studies have demonstrated the instrument’s validity, reliability, and salience [31]. Therefore, the objective of this study was to assess a modified Hennessey-Hicks Training Need Analysis instrument’s ability to measure the influence of an RN’s performance of social, technical, and socio-technical care tasks on length of stay in the CHF hospitalizations of African Americans.

**Table 1.** Data types, measures, and examples used in reducing mortality and lengths of stay

Variables	Types of data	Examples of measures	Examples of validated instruments and data collection tools
Anatomy and physiology	Laboratory values, panels, and imaging data	Glucose, lipid, systolic, diastolic panels, ejection fraction, B-type natriuretic peptide, pulmonary artery pressure	Complete Blood Count, Fasting Lipid Profile, Serum Electrolytes, Echocardiogram
Morbidity	Comorbidity and multimorbidity data	Presence and profiles of specific morbidities and combinations	Charlson Comorbidity Index, Adjusted Clinical Groups, Van Walraven Elixhauser Comorbidity
Clinical assessment	Clinical data	Vital signs, breathing assessment, fluid retention, physical assessment, mobility assessment	Weighing Scales, Body-Mass Index, Edema Index, New York Heart Association Functional Classification, Get with the Guidelines Heart Failure Risk Score
Medications	Medications, adherence, and management data	Patient confidence in medication management, forgetfulness, and regimen	Medication Adherence Self-Efficacy Scale, Morisky Scale, Adherence to Refills and Medications Scale
Self-management	Patient behaviors and experiences data	Cardiac diet, physical activity, recognition of changes in symptoms, preventative care, and confidence in self-care	Dietary Inflammation Index, Self-Care of Heart Failure Index, Patient-reported Outcome Measures Tool
Patient	Demographic and social determinants of health data	Education, employment, economic stability, housing, food, security, transportation, violence, and safety	Protocol For Responding to and Assessing Patient Assets, Risks, And Experiences Tool, Medical-Legal Advocacy Screening Questionnaire, Income, Housing, Education, Legal Status, Literacy, Personal Safety Questionnaire
Healthcare system	Organization determinants of care data	Organizational structures, climate, resources, patient safety, teamwork, expertise, job satisfaction, and electronic health record feature availability	Hospital Survey on Patient Safety Culture, the Teamwork and Safety Climate Survey, Assessment of Chronic Illness Care Tool, Practice Environment Scale of the Nursing Work Index

## Materials and methods

This study was reported in accordance with the guidelines for the consensus-based standards for the selection of health status measurement instruments (COSMIN) checklist [32].

### Study design

The study applied an observational, cross-sectional survey design using retrospectively collected EHR data and a quantitative job analysis survey. Based on EHR data, a total of 3498 RNs provided care to 22,703 patients with CHF within 113,543 CHF hospitalizations between January 1, 2015, and January 1, 2024. To test the psychometric properties of a modified Hennessey-Hicks Need Analysis instrument, a sample of 200 RNs were surveyed with respect to the outcomes of their patients. The study was conducted on the main campus of an academic health center in Arkansas, which is a primarily rural state. The hospital participates in the American Heart Association’s GWTG-HF initiative for implementing GDMT. The hospital has 535 beds, of which patients from all 75 counties of the state were provided care, including patients from counties with heart failure-related death rates that are twice the national average [15].

### Ethical considerations

The study protocol (#276211) was approved by the Institutional Review Board at the University of Arkansas for Medical Sciences. The informed consent process was conducted via email. The document included a basic description of the study purpose, survey completion time, data storage and retrieval processes, and contact information for the principal investigator. A waiver of documentation of the informed consent process was granted by the Institutional Review Board because the study presented only a minimal risk for loss of confidentiality.

### Sampling size and methods

**Figure 1.**
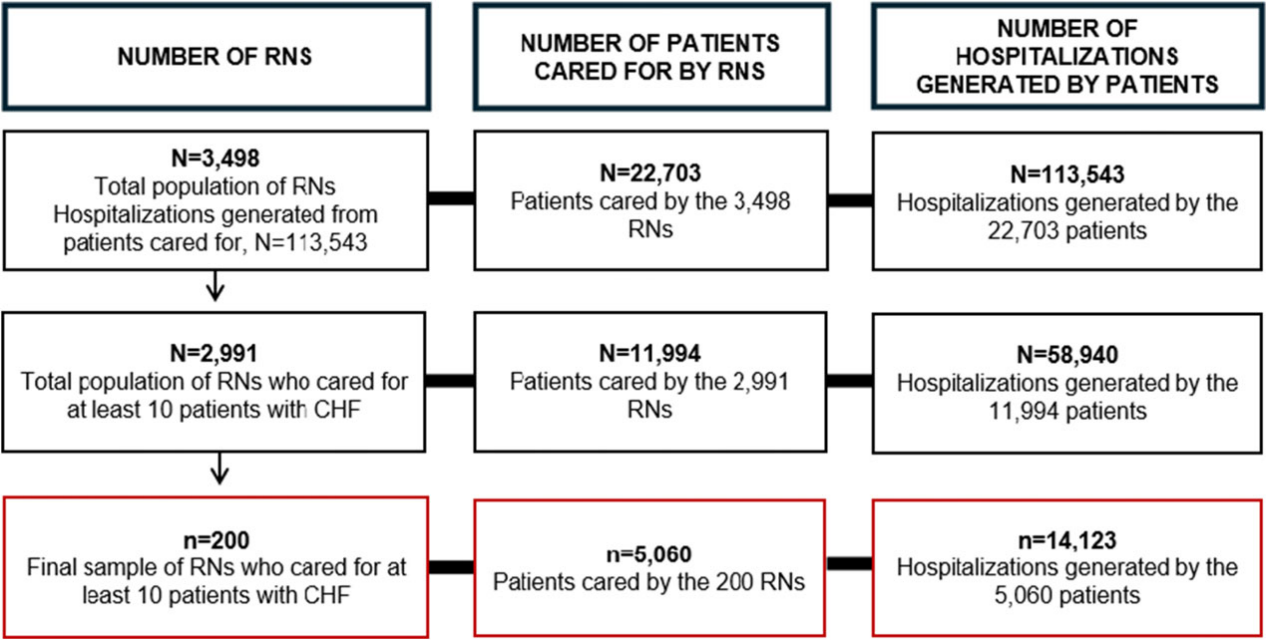
Flow diagram of sampling size. RNS = registered nurses; CHF = congestive heart failure.

A dataset was provided to the study team by the Arkansas Clinical Data Repository. It contained a de-identified list of African American patients with a primary diagnosis of CHF, length of stay, and an edge list containing pseudonyms that linked patients with the RNs who provided their care [14]. Stratified random sampling was used to select 100 RNs from above and 100 RNs from below the mean length of stay of the patients cared for by the total population of RNs. Stratified random sampling was applied to increase the representation of the RNs from the population and the likelihood of selecting RNs from the full distribution of patient length of stay. Based on EHR data, RNs who cared for fewer than 10 African Americans with CHF were excluded to minimize the selection of RNs who lacked sufficient experience in CHF care. Figure 1 shows how the inclusion criteria impacted the sample size.

### Instrument development and pretesting

Social, technical, and socio-technical task performance was captured via a modified Hennessey-Hicks Need Analysis instrument. The standard instrument contained 30 items that represent tasks performed during high-quality nursing care delivery [31]. However, the standard instrument was modified because it only had a minor focus on socio-technical tasks, and specificity was added to tailor it to assess clinical information use during the care of African Americans with CHF.

Sittig and Singh’s Socio-technical Framework for Health Information Technology served as the conceptual framework for the study [33–34]. The framework is focused on understanding how RNs work with data, information, and technology within its eight interdependent dimensions: 1) hardware and software computing infrastructure, 2) clinical content, 3) human-computer interfaces, 4) people, 5) workflows and communication, 6) internal organizational features, 7) external rules and regulations, and 8) measurement and monitoring [33–34]. The framework doesn’t conceptualize nursing care as a simple set of tasks, but as a set of dynamic interactions between people and processes within a complex adaptive system [33–34]. The framework was chosen because it was the most comprehensive conceptualization of the social, technical, and socio-technical processes of high-quality nursing care [33].

The modifications of the 30 items were guided by the study’s conceptual framework, and made within the modification requirements described in the Hennessey-Hicks Need Analysis manual [31,33–34]. For example, Item #1 of the standard instrument stated: “Establishing a relationship with patients.” The item was modified to state: “Using the EHR to establish a relationship with African American patients who have CHF.” Eight of the items in the standard instrument were removed because they did not align with the role of acute care RNs as articulated by the American Association of Heart Failure Registered Nurses’ Scientific Statement of the Nursing Care of Patients Hospitalized with Heart Failure [17]. These modifications were permitted by the manual of the standard Hennessey-Hicks Need Analysis instrument without compromising its psychometric properties [31]. A set of 22 items was retained in the final instrument, now constituting the Needs Analysis for Socio-technical Tasks, which represented three subscales: social, technical, and socio-technical tasks performed by RNs in the provision of CHF care. Of the 22 final items in the Needs Analysis for Socio-technical Tasks, Items 1–14 were modified from the basic items of the standard instrument. Items 15–22 were directly translated from all eight dimensions of Sittig and Singh’s Socio-technical Framework for Health Information Technology [31,33–34]. The Needs Analysis for Socio-technical Tasks was also pretested for content validity, usability, and technical functionality in an independent group of five acute care RNs who reflected the sampling frame of the target population of RNs.

### Measurement process

A closed survey approach was employed electronically through the Research Electronic Data Capture System between November 15, 2024, and January 31, 2025 [35]. Survey prompts discouraged all RNs from focusing on any specific EHR product, type, or company. Email addresses were used to manage reminders and survey completion. Demographic variables collected using the instrument included biological sex, race, ethnicity, and the number of years working as an RN. The RNs self-rated their performance in all 22 items (tasks) on a seven-point Likert scale (1–7), with higher ratings indicating higher socio-technical performance.

### Study variables

The primary study outcome was the length of stay (days). To preserve precision in the outcome measure, it was analyzed as a continuous measure and not rounded to the nearest integer. The study collected two types of independent variables: RN variables and variables on their patients. The primary independent variable was the total socio-technical performance score of the RN. The total socio-technical performance score was computed by summing the RN’s ratings of their own performance in the 22 items on the Needs Analysis for Socio-technical Tasks. Other covariates related to RNs were their years of experience, biological sex, race, and ethnicity. The patient-level covariates included in the analysis were age (years), biological sex, and in-hospital mortality. The health severity of patients was assessed via the Van Walraven Elixhauser Comorbidity Score (ranging from -19 to 89), with higher scores representing poorer health [14–16]. The score is a reliable predictor of increased LOS and in-hospital mortality in African American patients with CHF [14–16].

### Descriptive analysis

Open Refine 2.0 was used to confirm that there was no missing data for any study variables. The data was analyzed using SAS Version 9.4. All continuous variables were analyzed using descriptive statistics. The continuous variables were mean, standard deviation, median, inter-quartile range, minimum values, and maximum values. All categorical variables were presented using frequencies (percentages).

### Reliability analysis of the instrument

Classical test theory guided the statistical analysis of determining the extent to which the set of 22 socio-technical items measured the same domain [36–38]. Within classical test theory, item reliability identified items that were able to distinguish between an RN’s task performance and poorly designed items [36–38]. Item reliability was represented by corrected item-total correlations that identified the strength of the relationship between each item and the total socio-technical performance score [37]. Corrected item-total correlations below 0.15 are deemed unreliable, and those between 0.15 and 0.30 should be reviewed by experts (bedside RNs) for potential removal [36–38]. The internal consistency reliability of the instrument was estimated using Cronbach’s alpha. The Cronbach’s alpha correlated each of the RN’s ratings of the 22 items with their total socio-technical performance score on the instrument. The 95% confidence interval for Cronbach’s alpha was computed using a non-parametric bootstrapping approach with 10,000 bootstrap samples. Cronbach’s alpha was additionally estimated based on the deletion of each item to assess the impact of removing each item on the instrument’s reliability.

### Validity analysis of the instrument

A histogram assessed and visualized the distribution of length of stay. Length of stay was highly skewed and showed a notable departure from normality. Data transformation approaches (i.e., log transformation, square root transformation, and cube root transformation) failed to eliminate the skewness in the data. Therefore, to assess the criterion predictive validity of the Needs Analysis for Socio-technical Tasks, a generalized linear regression with a Gamma probability distribution and log-link function was used to model the association between total socio-technical performance score and length of stay. To account for the within-patient correlation in the study outcome, the Gamma regression models were fitted using generalized estimating equations. Regression modeling was done sequentially to obtain both unadjusted and adjusted measures of association. In step one, the length of stay was regressed on the socio-technical performance score. In step two, the model from step one was adjusted for the following variables: patient age, patient sex, patient Van Walraven Elixhauser Comorbidity Score, RN race, RN sex, and RN years of experience. The change in length of stay was estimated by back-transforming (exponentiating) the regression coefficients from the Gamma regression model. A two-sided *P*-value of 0.05 was used to determine statistical significance, and uncertainty in parameter estimates was reported using a two-sided 95% confidence interval.

## Results

A total of 200 RNs responded to all items on the instrument (100% response rate). The 200 RNs provided care to 5060 African American patients during 14,123 hospitalizations between January 1, 2015, and January 1, 2024. There was no missing data.

### Descriptive analysis results

Females were the largest sex in the sample and accounted for 82% (163 of 200) of RNs. Males accounted for 17% (34 of 200) of RNs. Approximately 2% (3 of 200) were of an unknown sex. Caucasians were the largest racial group of RNs, 72% (144 of 200). Native Hawaiian and other Pacific Islanders were the smallest racial group, accounting for less than 1% (1 of 200) of the sample. African Americans, Asian Americans, and American Indian and Alaskan Natives accounted for 15% (30 of 200), 6% (11 of 200), and 1% (2 of 200), respectively. Approximately 6% of RNs were of an unknown race (12 of 200). Individuals of Hispanic and Latino ethnicities accounted for approximately 5% (9 or 200) of RNs in the sample. Individuals of non-Hispanic and non-Latino ethnicities accounted for 92% (184 of 200) of RNs in the sample. Approximately 4% of RNs were of an unknown ethnicity (7 of 200). The mean number of years of experience working as an RN was 10.68 years (SD = 7.78). The mean number of years working with patients who had a chronic illness as an RN was 10.13 years (SD = 7.46).

The mean number of CHF patients cared for as an RN was 319 patients (SD = 329) (Table 2). The number of hospitalizations per patient ranged from 1 to 37. Female patients accounted for 2817 of the 5060 (56%) patients cared for by the 200 RNs, while males accounted for 2243 of the 5060 (44%) patients. The mean length of stay for all CHF patients cared for by RNs in the sample was 9.77 days (SD = 3.19) (Table 2). The mean Van Walraven Elixhauser Comorbidity Score for all CHF patients cared for by nurses in the sample was 18.50 (SD = 12.37), which indicated a medium level of patient health severity.

**Table 2.** Characteristics of the RNs and their patients

Characteristic	Mean (standard deviation)	Median (interquartile range)	Minimum Value (maximum value)
Total socio-technical performance score	111.49 (23.76)	112.50 (33)	22–154
Number of congestive heart failure patients cared for as a registered nurse	319 (329)	218 (327)	10 (1995)
Number of years working as a registered nurse	10.68 (7.78)	8 (9)	1.50 (38)
Number of years working with patients who had chronic illnesses as a registered nurse	10.13 (7.46)	8 (8.25)	<1 (38)
Patient age (years)	63.75 (14.77)	65.00 (20)	20 (89)
Patient length of stay (days)	9.77 (3.19)	9.99 (4.13)	1.68 (18.24)
Patient Van Walraven Elixhauser Comorbidity Score (health severity)	18.50 (12.37)	17 (17)	−14 (66)

### Reliability analysis results

Table 3 presents descriptives on task performance. The total possible scores ranged from 22 to 154. The mean total socio-technical performance score on the Needs Analysis for Socio-technical Tasks was 111.49 (SD = 23.76) (Table 2). The mean task performance in the 22 items ranged from 3.87 (SD = 1.87) to 5.73 (SD = 1.87). The Cronbach’s alpha coefficient for the overall instrument was 0.95 (95% CI: 0.94-0.96) and remained approximately 0.95 (ranging from 0.947 to 0.953) if any of the 22 items were to be deleted. The corrected item-total correlations for each of the 22 items ranged from 0.44 to 0.80.

**Table 3.** Task performance score by item

Item	Socio-technical task	Subscale	Mean (standard deviation)	Corrected item-total correlation	Cronbach’s Alpha If deleted
*14 Tasks from the Hennessy-Hick needs analysis*					
**1**	Using the electronic health record system to establish a relationship with African American patients who have congestive heart failure	Socio-technical	5.43 (1.51)	0.56	0.951
**2**	Reading published research on African Americans with congestive heart failure	Technical	3.87 (1.87)	0.59	0.951
**3**	Caring for patients with congestive heart failure	Socio-technical	5.69 (1.30)	0.65	0.950
**4**	Caring for African American patients with congestive heart failure	Socio-technical	5.73 (1.36)	0.72	0.949
**5**	Giving patient education information to African American patients or their caregivers	Social	5.52 (1.47)	0.66	0.949
**6**	Drawing your own conclusions about how to use the electronic health record to care for African American patients	Technical	4.72 (1.60)	0.59	0.950
**7**	Using risk scores or other information from the electronic health record system to improve a patient’s health	Socio-technical	5.10 (1.54)	0.70	0.949
**8**	Undertaking health promotion and prevention tasks to care for African American patients with congestive heart failure	Technical	4.92 (1.56)	0.80	0.947
**9**	Assessing African American patients’ clinical needs using the electronic health record system	Socio-technical	5.23 (1.47)	0.77	0.948
**10**	Collecting relevant information on the social determinants of health (ex., education, health literacy, safe housing, access to nutritious food) from the electronic health record system	Socio-technical	5.18 (1.48)	0.61	0.950
**11**	Working as a member of a congestive heart failure patient’s care team	Social	5.43 (1.46)	0.76	0.948
**12**	Accessing clinical resources to care for your congestive heart failure patients	Technical	4.94 (1.57)	0.74	0.948
**13**	Personally coping with burnout in your clinical environment	Social	4.46 (1.73)	0.44	0.953
**14**	Managing your overall workload of patients with the electronic health record system	Socio-technical	5.45 (1.34)	0.49	0.951
*8 Tasks from Sittig’s socio-technical framework for health information technology*					
**15**	Working with hardware and software related to the electronic health record system to care for a patient with congestive heart failure	Socio-technical	4.99 (1.52)	0.70	0.949
**16**	Working with information related to a patient’s congestive heart failure in the electronic health record system (ex., laboratory results, discharge summaries, or radiographic images) to care for the patient	Socio-technical	5.58 (1.42)	0.72	0.949
**17**	Working with the design of the electronic health record system as you retrieve information to provide care to patients with congestive heart failure	Socio-technical	5.03 (1.49)	0.68	0.949
**18**	The training or performance of other people in your environment who use the electronic health record system	Socio-technical	4.91 (1.53)	0.69	0.949
**19**	Using current processes to share information that provides each congestive heart failure patient with the care they need at the time they need it	Socio-technical	5.02 (1.49)	0.79	0.948
**20**	Working within internal organizational policies, procedures, and culture related to the electronic health record system to care for patients with congestive heart failure	Socio-technical	4.91 (1.51)	0.72	0.949
**21**	Working with external laws, regulations, and requirements that constrain your ability to use the electronic health record system to prevent a congestive heart failure patient’s death	Socio-technical	4.57 (1.74)	0.68	0.949
**22**	Continuously evaluate the quality of care that results from your use of the electronic health record to provide care for patients with congestive heart failure	Socio-technical	4.87 (1.71)	0.74	0.948

### Validity analysis results

**Figure 2.**
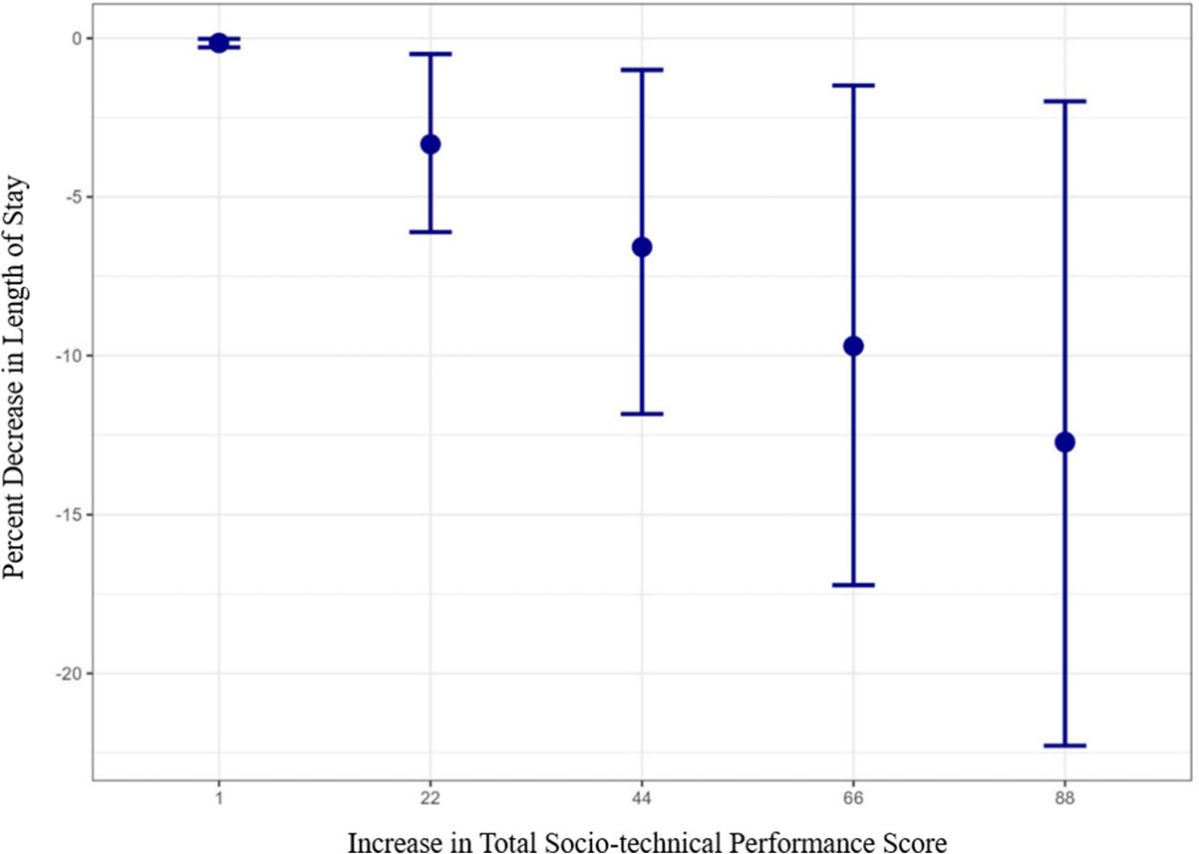
Adjusted estimated decrease in length of stay due to an increase in total socio-technical performance score.

Table 4 presents the results of the Gamma regression modeling, which showed a significant inverse association between an RN’s total socio-technical performance score on the Needs Analysis for Socio-technical Tasks and the length of stay in both unadjusted and adjusted analyses (*P*-value < 0.001). An increase in total socio-technical performance score of one point in the total score on the Needs Analysis for Socio-technical Tasks had an estimated reduction in length of stay of 0.15 (0.29,0.02) when adjusted. An increase of one to four points in each of the 22 items (totaling 22–88 points) was also presented in Table 4. For a one to four point increase in RN performance rating per item, the estimated reductions in the patient’s length of stay were 3.34% (6.11,0.5), 6.58% (11.84,1), 9.70% (17.22,1.49), and 12.72% (22.28,1.99), respectively (*P* = 0.004, Figure 2), when adjusted.

**Table 4.** Unadjusted and adjusted estimates of length of stay by increase in socio-technical performance score

Increase	Increase in mean total socio-technical performance score	Unadjusted estimates		Adjusted estimates	
		Percent decrease in length of stay (95% confidence interval)	*P*-value	Percent decrease in length of stay (95% confidence interval)	*P*-value
1-point total increase	1	0.19 (0.32,0.06)	0.004	0.15 (0.29,0.02)	0.004
1-point increase per item	22	4.05 (6.71, 1.32)	–	3.34 (6.11,0.5)	–
2-point increase per item	44	7.94 (12.96, 2.63)	–	6.58 (11.84,1)	–
3-point increase per item	66	11.67 (18.80, 3.92)	–	9.70 (17.22,1.49)	–
4-point increase per item	88	15.25 (24.24,5.19)	–	12.72 (22.28,1.99)	–

The regression was adjusted for RN race, RN years of work experience, patient age, patient sex, Van Walraven Elixhauser Comorbidity Score, and patient death. In the adjusted analysis, the Van Walraven Elixhauser Comorbidity Score and in-hospital mortality were also significantly associated with length of stay (*P*-value < 0.001; Table 5), indicating that patient health severity was associated with length of stay.

**Table 5.** Modeling of covariates in the adjusted estimates

Covariates	Estimates (95% confidence interval)	*P*-value
Registered nurse race: American Indian and Alaskan Natives	0.043 (−0.095,0.181)	0.542
Registered nurse race: Asian American	0.015 (−0.261,0.291)	0.916
Registered nurse race: African American	0.025 (−0.084,0.133)	0.654
Registered nurse race: other race	0.003 (−0.198,0.205)	0.975
Registered nurse race: Caucasian American (reference group)	–	–
Registered nurse years of experience	−0.001 (−0.013,0.012)	0.904
Registered nurse sex: male	0 (−0.121,0.121)	0.996
Registered nurse sex: unknown	−0.273 (−0.596,0.05)	0.097
Registered nurse sex: female (reference group)	–	–
Van Walraven Elixhauser Comorbidity Score (patient severity)	0.019 (0.015,0.023)	<0.001
Patient In-hospital death: yes	0.472 (0.371,0.573)	<0.001
Patient In-hospital death: no (reference group)	–	–
Patient age	−0.002 (−0.005,0.001)	0.26
Patient sex: male	0.018 (−0.065,0.1)	0.674
Patient sex: female (reference group)	–	–

## Discussion

While many factors influence length of stay, the study found that social, technical, and socio-technical care tasks performed by RNs may influence length of stay. The total socio-technical performance score of the Needs Analysis for Socio-technical Tasks was significantly associated with the length of stay in the CHF hospitalizations of African Americans. Reliability and validity demonstrated significant promise toward accounting for social, technical, and socio-technical care task performance in areas such as quality monitoring and improvement. The instrument can be used to measure, stratify, and control for the impact of nursing care on length of stay. This instrument may be particularly impactful in addressing problems with overfitting or model convergence by providing a single score to account for the influence of nursing care delivery compared to including all 22 items individually.

### Reliability of the instrument

The Cronbach’s alpha coefficient was 0.95 (95% CI: 0.94–0.96) and remained approximately 0.95 (ranging from 0.947 to 0.953) if any of the 22 items were deleted. This Cronbach’s alpha coefficient fell within the higher range of other published modifications of the instrument (i.e., 0.70–0.95) in areas such as community nursing and cancer care [31]. This indicated a high level of internal consistency reliability within the set of 22 items because it exceeded the established reliability threshold of 0.80 [38]. While a Cronbach’s alpha coefficient of 0.80 to 0.95 is ideal, coefficients above 0.95 may indicate highly correlated and repetitive items [38]. However, the coefficient of 0.95 indicated that the items on the instrument were a well-scoped measure of the same domain. The total set of 22 items was derived from multiple sources: the standard Hennessey-Hicks Need Analysis and Sittig and Singh’s Socio-technical Framework for Health Information Technology. As a result, the items from each of the sources may have been loading more closely together by source due to an artifact of the original measurement design of each source [38]. This may have increased item cohesion and internal consistency by source, subscale, and/or in the overall set of items.

The corrected item-total correlations for each of the 22 items ranged from 0.44 to 0.80. These values indicated that all items were above the established thresholds for reliable items and distinguished an RN’s social, technical, and socio-technical task performance from poorly designed items that were not able to measure their performance (i.e. < 0.15) [36–38]. The results showed that even if the item with the lowest corrected item-total correlation (0.44) was removed from the instrument (Item 13), the Cronbach’s alpha coefficient would have only increased trivially by 0.003 (from 0.95 to 0.953). This indicated that no items should be removed because there was a general latent factor underlying all the items on the instrument [38]. Therefore, each item contributed meaningfully to the instrument’s ability to consistently measure the influence of social, technical, and socio-technical task performance. The range of the corrected item-total correlations also indicated that the instrument is multifaceted due to the existence of the three subscales within the instrument: social, technical, and socio-technical tasks [38].

### Validity of the instrument

While content validity was evaluated during pretesting, the study should be further validated in practicing acute care RNs (i.e., excluding nurse managers without an active caseload) to reduce the potential influence of unidentified confounding variables that may have been unique to the environment of the one hospital that was surveyed in this study. These additional studies of content validity should also employ techniques such as keystroke modeling, task mapping, and task decomposition analyses for each of the 22 tasks. This will give consideration to the complex adaptive systems of nursing care and its seamless interaction with the workflows and multidisciplinary tasks performed by other disciplines in CHF care. These methods will also identify the specific elements of tasks (e.g., time spent performing the tasks, subtasks, variation in approaches) that contribute to optimization and reductions in length of stay.

Criterion predictive validity indicated a high degree of association between the total socio-technical performance score and the length of stay of African American patients (*P* = 0.004) [38–39]. The regression found that an increase in total socio-technical performance score of one to four points in each of the 22 socio-technical performance items would result in a decreased length of stay of 3.34% (6.11,0.5), 6.58% (11.84,1), 9.70% (17.22,1.49), and 12.72% (22.28,1.99), respectively. For example, for an RN who cared for an African American patient who had a total length of stay of 3 days (i.e., 72 hours) and whose initial total socio-technical performance score on the instrument was 100, an increase in total socio-technical performance score of 22 points was associated with a 3.34% (i.e., approximately 2 hours and 40 minutes) decrease in the patient’s length of stay. A potential 2-hour and 40-minute decrease in length of stay is clinically meaningful, particularly in hospitals like the one surveyed here, whose capacity is exacerbated by the highest geographic concentrations of CHF prevalence, death rates, and mortality risk that are nearly twice the national average [15]. It is anticipated that the specific resulting decrease in length of stay may vary, as model prediction is specific and sensitive to the self-reported performance of the RN. This sensitivity is a strength of the instrument because it addresses national calls to establish a real-time feedback and performance improvement measure of the dynamic nature of nursing care delivery (if embedded and automated within EHRs through existing processes such as the nursing checklists of CHF-specific units) [9]. However, like all data and instruments that have been integrated into clinical workflows, workflow optimization studies should be conducted to ensure the effective translation of the instrument. For example, if integrated into a nursing checklist during the provision of care and RNs are surveyed every shift, it may be simply viewed as just another unimportant task unrelated to their direct provision of care. As a result, response bias could be introduced (e.g., an RN just clicking through the survey to get it done), which would decrease the accuracy of the instrument’s prediction. Workflow optimization studies could address any nuances related to the instrument’s implementation. For example, these studies could assess deployment within EHRs or algorithms to identify scores that are outside the established thresholds for a hospital, unit, or the normal range of a specific RN’s score history (i.e., to detect response bias).

### Translation and optimization of guideline-directed medical therapy

The use of the instrument, along with appropriate workflow optimization and training on its use, operationalizes the measurement of clinical information use in GDMT, which is critical to caring for African Americans with CHF. For example, social determinants of health are responsible for 25.8% of the risk for long lengths of stay in African American patients compared to only 10.1% in non-African Americans [7]. While there are many instruments to collect social determinants of health data from patients, they do not measure the effectiveness of the collection process itself, which is critical to ensuring the translation and use of the information [7,18]. However, our findings provide a validated instrument for measuring the influence of an RN’s collection of social determinants of health information in nursing care (Item 10).

In the adjusted analysis, the Van Walraven Elixhauser Comorbidity Score (health severity) and in-hospital mortality were significantly associated with length of stay, which is also consistent with recent findings in nursing care [14,27]. This finding indicated that the performance of RNs alone is generally not the only influence on length of stay. Therefore, the Needs Analysis for Socio-technical Tasks should be used in conjunction with other instruments and types of data used in CHF care (i.e., ranging from anatomical and physiological data to healthcare system data) to comprehensively measure, stratify, predict, and control for all influences on length of stay [9,13,18–29]. The instrument’s strong validity and reliability present an opportunity to establish the 22 items as national quality measures of nursing care within initiatives such as the American Heart Association’s GWTG-HF [8,9,14,27]. Subsequently, structured interventions and toolkits should be developed to address and increase the performance of RNs, by task, which could reduce the lengths of stay of patients. For example, identifying and increasing performance in Item 10 (i.e., the collection of social determinants of health information) could decrease lengths of stay through interventions such as providing transportation vouchers for follow-up visits to a cardiologist, optimizing patients’ receipt of GDMT [7]. The instrument also measures information use in other areas unique to chronic disease care. These areas include assessing the use of patient data collected longitudinally, as the top 5% of patients with multiple chronic diseases have historically accounted for 50% of healthcare spending in the United States (Items 15,17,20,21,22), patient self-management (5 and 8), and assessing the influence of care delivery (Items 3,4,12,13,14) [11,17,27,40,41]. The instrument could improve prescribing within GDMT by assessing the use of longitudinal data related to searching for and importing clinical data into notes, visualizing trends in laboratory values and previous medications, and triggering clinical recommendations [13]. For example, the instrument could assess an RN’s use of a structured, CHF-specific note that was prepopulated with trends in laboratory values [13]. For hospitals whose GDMT is formally guided by GWTG-HF, the instrument further facilitates the use of information related to: the evaluation of heart function (Items 9 and 16), using information based on evidenced-based care (Items 2 and 7), structured discharge CHF education for self-care (Items 5 and 8), and care communication and coordination processes such as scheduling follow-up visits within 72 hours of discharge (Items 1,11,18,19) [11].

### Limitations

The study is based on a single academic health center in a rural state and may not reflect the structure of nursing care delivery at all hospitals in the United States. This may limit the generalizability of the findings and the instrument to only RNs, African American patients, and hospitals in the United States with similar characteristics as the RNs, African American patients, and hospitals presented in this study. Additionally, the Van Walraven Elixhauser Comorbidity Score was used as the sole measure of patient health severity. There are many additional indicators of health severity, including left ventricular ejection fraction, that may be appropriately applied to strengthen the representation of a patient’s health severity. Based on the study design, the instrument provides a nursing-based self-assessment of their own performance, which could be perceived as subjective in nature. As a result, the instrument should be used in collaboration with established objective measures of care in GDMT, such as defect-free care for quadruple therapy for patients with reduced ejection fraction [8,9]. Further research is needed to determine the specific proportion of weight that should be placed on subjective and objective measures within clinical risk stratification processes and adjustments to data analyses. This will ensure the effective accounting of and cohesiveness with other factors, such as heart function, that may more directly influence length of stay.
